# Mindfulness-based group therapy for inpatients with schizophrenia spectrum disorders – feasibility, acceptability, and preliminary outcomes of a rater-blinded randomized controlled trial

**DOI:** 10.1192/j.eurpsy.2021.2130

**Published:** 2021-08-13

**Authors:** K. Böge, I.M. Hahne, N. Bergmann, M. Zierhut, T.M.T. Ta, K. Wingenfeld, M. Bajbouj, E. Hahn

**Affiliations:** Department Of Psychiatry, Charité – Universitätsmedizin Berlin, Campus Benjamin Franklin, Berlin, Germany

**Keywords:** randomized controlled trial, Schizophrenia spectrum disorders, mindfulness, psychotherapy

## Abstract

**Introduction:**

The therapeutic effectiveness of mindfulness-based interventions (MBIs) has been shown for various mental disorders. However, for schizophrenia spectrum disorders (SSD), only a few trials have been conducted, mostly in outpatient settings.

**Objectives:**

This study aimed to investigate feasibility, acceptability, and preliminary effectiveness of a four-week mindfulness-based group therapy (MBGT) for in-patients with SSD.

**Methods:**

A pre-registered randomized controlled trial (RCT) was conducted at the in-patient ward for SSD. All measures were employed at baseline, post-intervention (4-weeks), and follow-up (12-weeks). The primary outcome was ‘mindfulness’. Secondary outcomes were rater-blinded positive- and negative symptoms, depression, social functioning, as well as self-rated mindfulness, depression, anxiety, psychological flexibility, quality of life, and medication regime.

**Results:**

N=40 participants were randomized into either four-week treatment-as-usual (TAU; n=19) or MBGT+TAU (n = 21). Protocol adherence was 95.2%, and the retention rate to treatments was 95%. ANCOVA analysis revealed significant improvements in the MBGT+TAU compared to TAU for the primary outcome and negative symptoms. Exploratory analyses showed medium-to-large intervention effects on secondary outcomes mindfulness, positive, negative, and depressive symptoms, psychological flexibility, quality of life, and social functioning for MBGT+TAU and small-to-moderate changes on positive symptoms and social functioning for TAU. No serious adverse effects were reported.

**Conclusions:**

This study supports the feasibility and acceptability of MBGT for in-patients with SSD, including high protocol adherence and retention rates. A proof of concept of the MBIs and corresponding improvements on various clinical and process parameters warrant a fully powered RCT to determine effectiveness, cost-efficiency, and longitudinal outcomes of MBGT for SSD.

**Disclosure:**

No significant relationships.

